# Supplementary material for: Performance of SARS-CoV-2 serology tests: Are they good enough?
Source: PLoS One. 2021 Feb 17;16(2):e0245914. doi: 10.1371/journal.pone.0245914 (PMC7888604; doi:10.1371/journal.pone.0245914)
Supplement: Attachment — Submitted filename: Review PONE-D-20-31021-11-6-20.docx [file pone.0245914.s002.docx]

Major comment: The manuscript# PONE-D-20-31021entitled “Performance of SARS-CoV-2 Serology tests: Are they good enough?” assess the performance of SARS-CoV-2 serologic tests available in clinical practices.

These methods were introduced into clinical practice quickly without an extensive validation usually required by the regulatory authorities. The IgG serologic Assays from EuroImmun, Epitope Diagnostics (EDI), Abbott Diagnostics and DiaSorin and a rapid IgG/IgM test from Healgen were evaluated for the imprecision, diagnostic sensitivity and specificity and concordance. The SARS-CoV-2 patient samples were analyzed as pre-pandemic control specimen with confirmed respiratory infections, inflammatory polyarthritis and several samples positive for thyroid stimulating immunoglobulin and post pandemic samples. Authors showed that the diagnostic sensitivity of the assays were 100% (CI: 80-100%) for Abbott, EDI and EuroImmun and 95% (CI: 73-100%) for DiaSorin at ≥14 days post infection. Only the Abbott assay had a diagnostic specificity of 100% (CI: 91-100%). The DiaSorin displayed more false negative results across all conditions tested. The imprecision for EDI and EuroImmun, was 0.02-14.0% CV while for Abbott and DiaSorin imprecision (CV) ranged from 5.2% - 8.1% and 8.2% - 9.6% respectively. Overall, concordance of the assays ranged from 76.1% to 97.9%. The point of care rapid test Healgen, showed excellent sensitivity and specificity. Authors attributed the differences in results to the use of nucleocapsid and spike proteins. The point of care device tested demonstrated adequate performance for antibody detection.

Limited sample size should be included in the limitation of the study.

The Figure legend describes Figure 1 as Box-plot even though data shown as a dot plot.

The Figure 1A reveled very little to no cross reactivity. The statistical analysis among groups show significant differences between N to RA and N to TSI. None of the values in RA and only one value in TSI groups is above the positive cutoff value. May be author should comment on meaning of such statistical significance.

In this study the DiaSorin displayed the most-false negatives and showed cross-reactivity when comparing with other serologic assays. It will important to add in the discussion the frequency of use of these tests within UK or globally specially if that data is available through health care systems or through the company.
