# Supplementary material for: Performance of SARS-CoV-2 serology tests: Are they good enough?
Source: PLoS One. 2021 Feb 17;16(2):e0245914. doi: 10.1371/journal.pone.0245914 (PMC7888604; doi:10.1371/journal.pone.0245914)
Supplement: Attachment — Submitted filename: Response to Reviewers.docx [file pone.0245914.s003.docx]

**Dear Editor**	*December 2020*

Concerning manuscript ID**:** PONE-D-20-31021

Title: Performance of SARS-CoV-2 Serology tests: Are they good enough?

Thank you for your willingness to reconsider our manuscript if it is revised according to the reviewers' comments. The authors are extremely grateful for the reviewer’s time and extensive and constructive comments. Below you will find point-by-point answers to questions raised. In the “Revised Manuscript”, the changes to the original manuscript are highlighted in YELLOW. Changes to the formatting have been tracked using word tracking system only. A revised manuscript with no tracking is also submitted. We refer to the changes here by line of appearance. The Cover letter is also resubmitted with the changes requested by the editorial team.

The co-authors have consented to the changes of the manuscript.

**Editor's Comments:**

Please remove any funding-related text from the manuscript and let us know how you would like to update your Funding Statement. Currently, your Funding Statement reads as follows: [The funders had no role in study design, data collection and analysis, decision to publish, or preparation of the manuscript.]

**Answer**: We have removed the statement from the acknowledgement section and revised the cover letter to reflect the changes.

Please include captions for your Supporting Information files at the end of your manuscript, and update any in-text citations to match accordingly. Please see our Supporting Information guidelines for more information: [http://journals.plos.org/plosone/s/supporting-information](about:blank).

**Answer**: A section Supporting Information has been added at the end of the manuscript line 519

**Reviewer#1**

Major comment: The manuscript# PONE-D-20-31021entitled “Performance of SARS-CoV-2 Serology tests: Are they good enough?” assess the performance of SARS-CoV-2 serologic tests available in clinical practices.
These methods were introduced into clinical practice quickly without an extensive validation usually required by the regulatory authorities. The IgG serologic Assays from EuroImmun, Epitope Diagnostics (EDI), Abbott Diagnostics and DiaSorin and a rapid IgG/IgM test from Healgen were evaluated for the imprecision, diagnostic sensitivity and specificity and concordance. The SARS-CoV-2 patient samples were analyzed as pre-pandemic control specimen with confirmed respiratory infections, inflammatory polyarthritis and several samples positive for thyroid stimulating immunoglobulin and post pandemic samples. Authors showed that the diagnostic sensitivity of the assays were 100% (CI: 80-100%) for Abbott, EDI and EuroImmun and 95% (CI: 73-100%) for DiaSorin at ≥14 days post infection. Only the Abbott assay had a diagnostic specificity of 100% (CI: 91-100%). The DiaSorin displayed more false negative results across all conditions tested. The imprecision for EDI and EuroImmun, was 0.02-14.0% CV while for Abbott and DiaSorin imprecision (CV) ranged from 5.2% - 8.1% and 8.2% - 9.6% respectively. Overall, concordance of the assays ranged from 76.1% to 97.9%. The point of care rapid test Healgen, showed excellent sensitivity and specificity. Authors attributed the differences in results to the use of nucleocapsid and spike proteins. The point of care device tested demonstrated adequate performance for antibody detection.

Limited sample size should be included in the limitation of the study.

**Answer**: We added a section “strengths and limitations of the study” line 376-384 commenting on the limited number of samples and the fact we had no access to symptoms onset or severity. We also commented on line 376-385 that we chose to use only one data point per patient to avoid any bias due to repeat measurement. Time course analysis was done separately.

The Figure legend describes Figure 1 as Box-plot even though data shown as a dot plot.

**Answer**: We thank the reviewer for pointing this error to us; this has now been corrected line 233.

The Figure 1A reveled very little to no cross reactivity. The statistical analysis among groups show significant differences between N to RA and N to TSI. None of the values in RA and only one value in TSI groups is above the positive cutoff value. May be author should comment on meaning of such statistical significance.

**Answer**: We addressed this point line 341-344. It reads:

” Using the EDI assay, the signal obtained for both RA and TSI samples is significantly higher than the negative controls however all but one TSI sample remain below the cut-off of positivity. The cut-off is therefore appropriate for use with the assay with potentially cross-reactive substances”

In this study, the DiaSorin displayed the most-false negatives and showed cross-reactivity when comparing with other serologic assays. It will important to add in the discussion the frequency of use of these tests within UK or globally especially if that data is available through health care systems or through the company.

**Answer**: The authors agree fully with the reviewer. We have updated the manuscript line 367-372 as follow: “In April 2020 both the DiaSorin assay and the Abbott assays were authorised by Public Health England for emergency use in the clinical setting; 15 sites using the Abbott method reported to the WEQAS scheme in October while 84 (Abbott Architect and Alinity) reported to the UKNEQAS scheme in November. However, 3 sites using the DiaSorin method reported to WEQAS and 9 to UKNEQAS for the same period.”

A link to the UKNEQAS scheme has also been added line 379 along with the WEQAS link.

**Reviewer #2:** This is a well written manuscript. COVID -19 is also a huge demanding topic. I have some minor comments.
Overall: The number of positive samples is very low (43). Though the present analysis showed a good agreement. But it may alter if the sample size increased. There are so many published paper with the information of different type of serological testing of SARS-CoV-2. Presently we need more specific decision for specific serological test.

Please add some explanation in discussion section, why the specificity of DiaSorin assay showed low.

**Answer**: We observe a lower specificity of the DiaSorin assay as compared to the Abbott and EDI. The authors advanced the hypothesis that this difference may be due to the different design, DiaSorin (and Euroimmun) using spike protein while Abbott and EDI use the nucleocapsid (line 354-359) however there may be other factors influencing the results (choice of enzyme conjugate, etc). It is evident that the sample size here is low, however not biased by using different time points of the same patients. As the pandemic runs its course, the number of patient’s samples available will unfortunately increase. Systematic reviews will also need to be performed to pull the data together from worldwide studies to determine whether some assays give different results depending on the cohort studied (geographical variance in the virus), giving deeper understanding of the assay differences. As we resubmit this manuscript, the UK health authorities have declared the identification of a new variant of the virus. More information will be therefore growing to answer this question.

Line 111: Please add the time period of the SARS-CoV-2 positivity; like March to May.

**Answer**: The samples were collected between April and beginning of June; this information has now been added line 115.

**Reviewer #3:**

The study is well designed and results are clear presented.
Concerning the results higher sensitivity for point of care devices was found, compared with other publications. The absence of cross-reactivity to seasonal coronavirus and influenza A is a remarkable result. Evolution of the virus and antibodies production turns serology assays a complex and hard work.

Although Authors did not consider strengths or weaknesses of the work.

**Answer**: A section “strengths and limitations of the study” was added line 383-392 commenting on the limited number of samples and the fact we had no access to symptoms onset or severity. We also commented on line 301-302 that we chose to use only one data point per patient to avoid any bias due to repeat measurement. Time course analysis was done separately

To date the samples for serology PCR day was used, but it would be better to date from onset of symptoms.
The study is small and results probably will need to be reproduced in larger studies, in different places and in systematic reviews.

**Answer**: As we commented on the strength and limitations section, we did not have access to the symptoms onset and severity, all patients were in hospital when diagnose, however hospitalisation may not have been due to COVID in the first place..

Line 268 – we chose – please correct.

**Answer**: We replaced the word chose by analyzed line 318.
